# Supplementary material for: Divergent molecular signatures in fish Bouncer proteins define cross-fertilization boundaries
Source: Nat Commun. 2023 Jun 14;14:3506. doi: 10.1038/s41467-023-39317-4 (PMC10267171; doi:10.1038/s41467-023-39317-4)
Supplement: Supplementary file 1 — Supplementary Information [file 41467_2023_39317_MOESM1_ESM.pdf]

Supplementary Information for

Divergent molecular signatures in fish Bouncer proteins define cross-fertilization boundaries

Krista R.B. Gert, Karin Panser, Joachim Surm, Benjamin S. Steinmetz, Alexander Schleiffer, Luca Jovine, Yehu Moran, Fyodor Kondrashov, and Andrea Pauli*

*Corresponding author: andrea.pauli@imp.ac.at

This PDF file includes:

Supplementary Figures 1-5

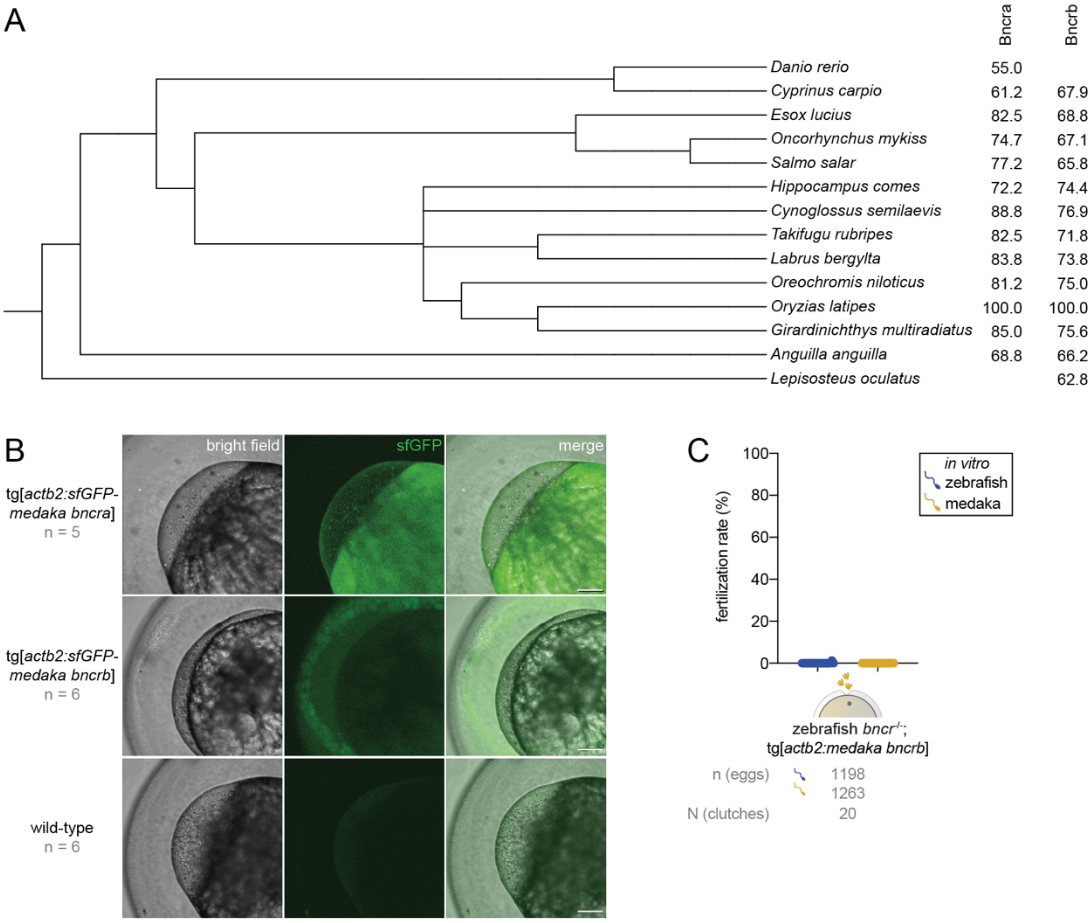

Supplementary Figure 1. Most fish encode two Bncr proteins, Bncra and Bncrb. (A) Taxonomic tree depicting the presence/absence of Bncra and Bncrb in selected fish species. All Bncr homologs of the respective species were assigned to either Bncra or Bncrb subfamilies based on the highest similarity to its medaka ortholog. **(B)** Confocal maximum intensity Z-projections of transgenic zebrafish *bncr*$^{-/-}$ eggs expressing sfGFP-tagged medaka Bncra (top) and Bncrb (middle). Wild-type zebrafish eggs with no transgene are shown below. Scale bar = 100 µm. **(C)** Medaka/zebrafish IVF with transgenic zebrafish *bncr*$^{-/-}$ eggs expressing sfGFP-tagged medaka Bncrb.

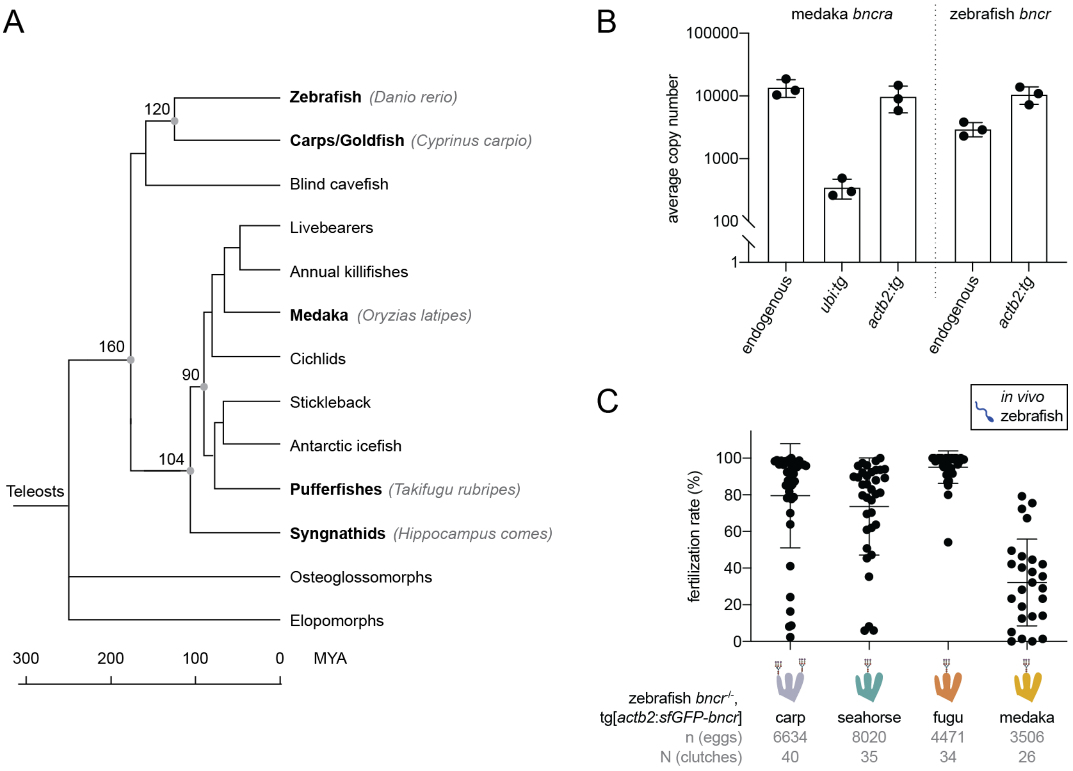

Supplementary Figure 2. Different species' Bncr proteins are compatible with zebrafish sperm *in vivo*. (A) Phylogeny and divergence times of fish species groups whose Bncr proteins were included in this study (bold) in addition to other teleost families for reference. Divergence times in MYA[1] are given for the nodes corresponding to the most recent common ancestor of the fish species whose Bncrs were tested. Figure adapted from[2,3]. **(B)** Average copy number of endogenous, *ubiquitin* promoter-driven, and *actin* promoter-driven medaka *bncra* in wild-type medaka eggs and transgenic zebrafish *bncr*$^{-/-}$ eggs as measured by qPCR (left). Average endogenous and *actin* promoter-driven copy numbers for zebrafish *bncr* in wild-type and transgenic zebrafish *bncr*$^{-/-}$ eggs, respectively, as measured by qPCR (right). Y-axis is plotted in $\log_{10}$ scale. All data were obtained from 3 biological replicates. **(C)** *In vivo* fertilization rates of transgenic zebrafish *bncr*$^{-/-}$ lines expressing carp, seahorse, fugu, and medaka *bncra*. Fertilization rates with an *actin* promoter-driven zebrafish *bncr* rescue line were previously reported[4]. Because each line may have a different expression level of its respective transgene vs. another, statistical comparisons between lines were not performed. Means ± SD are indicated in panels B and C.

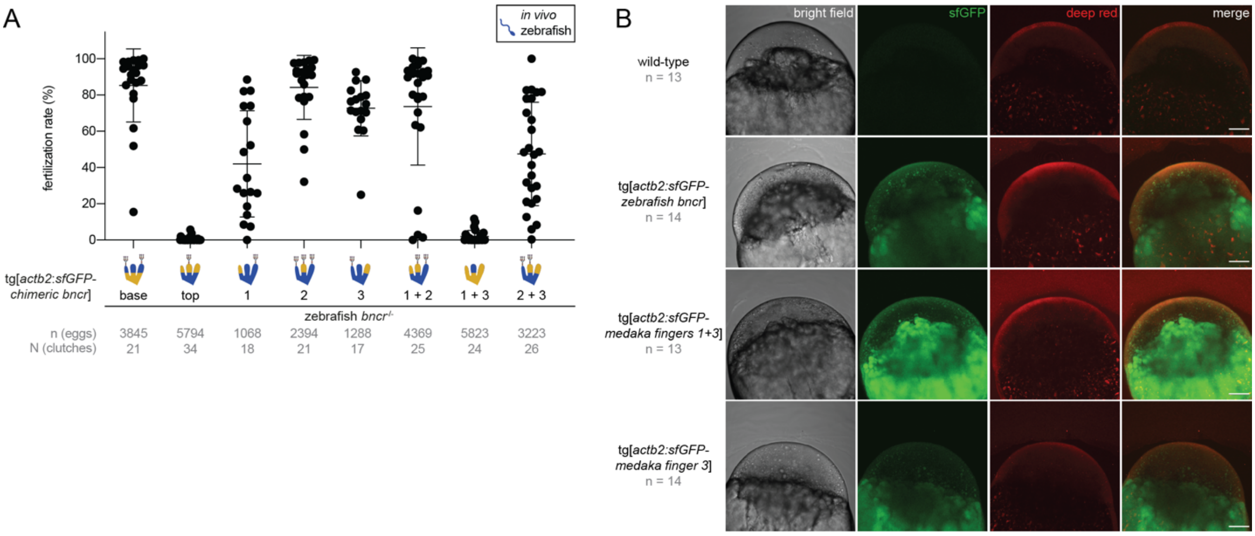

Supplementary Figure 3. Assessment of the compatibility of medaka/zebrafish Bncr chimeras with zebrafish sperm *in vivo*. (A) *In vivo* fertilization rates of transgenic zebrafish *bncr*$^{-/-}$ lines expressing sfGFP-tagged chimeric medaka/zebrafish Bncr constructs. The "finger(s)" or region(s) of zebrafish Bncr that were exchanged for that of medaka Bncr are indicated below the X-axis. Because each line may have a different expression level of its respective transgene vs. another, statistical comparisons between lines were not performed. Means ± SD are indicated. **(B)** Confocal maximum intensity Z-projections of wild-type (top) and transgenic zebrafish *bncr*$^{-/-}$ eggs (below). Chimeric sfGFP-tagged Bncr constructs medaka fingers 1 + 3 (3rd row) and medaka finger 3 (bottom row) show expression at the egg membrane, similar to zebrafish *bncr* (2nd row). Red, CellMask Deep Red membrane stain. Scale bar = 100 µm.

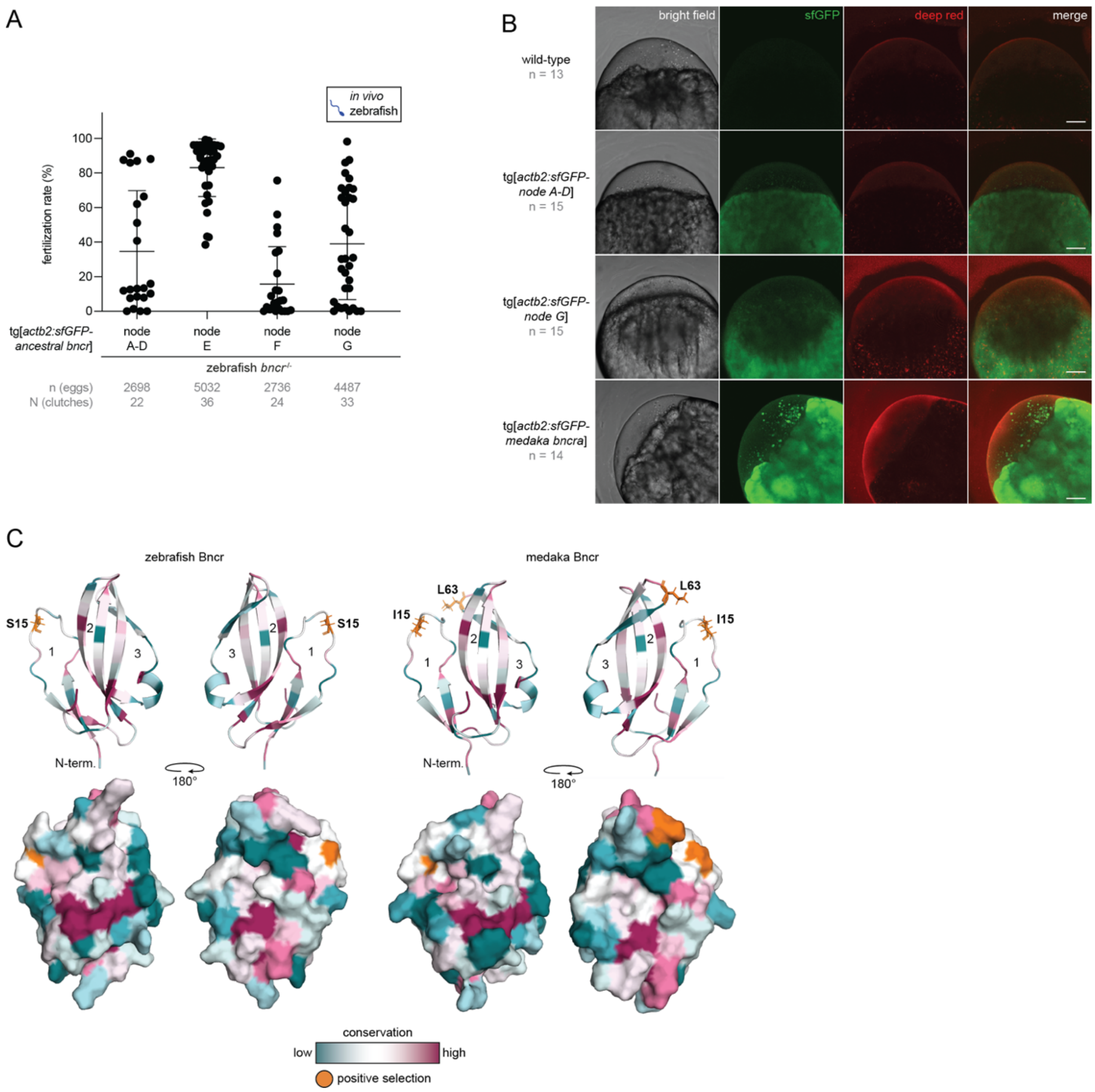

Supplementary Figure 4. Ancestral versions and positively selected amino acid sites in Bncr. (A) *In vivo* fertilization rates of transgenic zebrafish *bncr*$^{-/-}$ lines expressing sfGFP-tagged ancestral Bncr states at nodes A-D, E, F, and G. Because each line may have a different expression level of its respective transgene vs. another, statistical comparisons between lines were not performed. Means ± SD are indicated. **(B)** Confocal maximum intensity Z-projections of wild-type (top) and transgenic zebrafish *bncr*$^{-/-}$ eggs (below). Ancestral sfGFP-tagged Bncr constructs nodes A-D (2nd row) and node G (3rd row) show expression at the egg membrane, though are more weakly expressed than medaka *bncra* (bottom row). Red, CellMask Deep Red membrane stain. Scale bar = 100 µm. **(C)** Cartoon and surface representation models of zebrafish (left) and medaka (right) Bncr proteins predicted by AlphaFold[5,6] with sites colored according to conservation level. Site 15 is under positive selection in both zebrafish and medaka Bncr, whereas site 63 is under positive selection specifically in the medaka lineage. Amino acids in positively selected sites are colored orange; conservation level ranges from low (dark teal) to high (dark magenta).

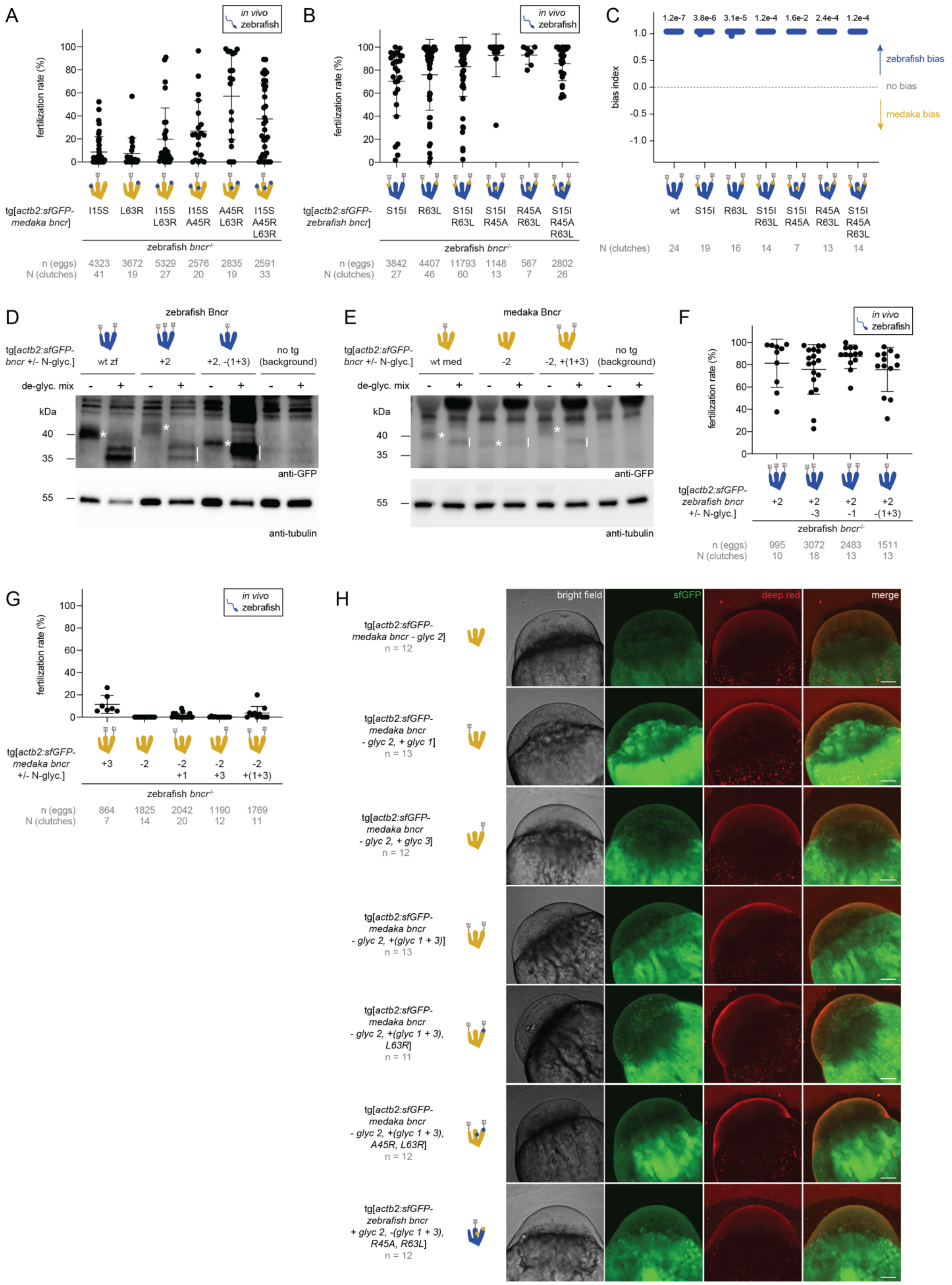

Supplementary Figure 5. Importance of specific amino acids and distinct glycosylation patterns in medaka and zebrafish Bncr. (A) *In vivo* fertilization rates of transgenic zebrafish *bncr*$^{-/-}$ lines expressing sfGFP-tagged medaka Bncr constructs with zebrafish amino acid

substitutions. Means ± SD are indicated. **(B)** *In vivo* fertilization rates of transgenic zebrafish *bncr*$^{-/-}$ lines expressing sfGFP-tagged zebrafish Bncr constructs with medaka amino acid substitutions. Means ± SD are indicated. **(C)** Bias index derived from IVF data in Fig. 5B. All constructs show bias for zebrafish sperm. (Two-tailed Wilcoxon signed rank test vs. theoretical median of 0 with the method of Pratt). **(D-E)** Western blot with GFP antibody of zebrafish Bncr (D) and medaka Bncr (E) N-glycosylation variant egg lysates, untreated vs. treated with de-glycosylation enzyme mix. A higher molecular weight (m.w.), glycosylated GFP-Bncr signal accompanied by a smear is visible in the untreated samples (*) which shifts downward to ~35 kDa upon de-glycosylation (|). Untreated sample bands show the highest m.w. for zebrafish Bncr with three N-glycosylation sites (+2) above 40 kDa, followed by ~40 kDa for two N-glycosylation sites (wt zf), and below 40 kDa for one N-glycosylation site (+2, -(1+3)), in line with all constructs being N-glycosylated as expected. The untreated medaka Bncr band with two N-glycosylation sites (-2, +(1+3)) runs highest above 40 kDa, followed by the band for untreated medaka Bncr with one N-glycosylation site (wt med). The bands from all de-glycosylated samples and untreated medaka Bncr with no N-glycosylation site (-2) run at the same height below 40 kDa. Wild-type zebrafish embryo lysate (no transgene) is shown to indicate background signal. Tubulin is shown as loading control; n = 1 for both blots. **(F)** *In vivo* fertilization rates of transgenic zebrafish *bncr*$^{-/-}$ lines expressing sfGFP-tagged zebrafish Bncr N-glycosylation variants. Means ± SD are indicated. **(G)** *In vivo* fertilization rates of transgenic zebrafish *bncr*$^{-/-}$ lines expressing sfGFP-tagged medaka Bncr N-glycosylation variants. Means ± SD are indicated. **(H)** Confocal maximum intensity Z-projections of transgenic zebrafish *bncr*$^{-/-}$ eggs expressing the indicated Bncr N-glycosylation and amino acid variants at the membrane. For positive and negative controls, see Suppl. Fig. 4B. Red, CellMask Deep Red membrane stain. Scale bar = 100 μm.

Supplementary References

1. Lin, Q. *et al.* The seahorse genome and the evolution of its specialized morphology. *Nature* **540**, 395–399 (2016).

2. Braasch, I. *et al.* A new model army: Emerging fish models to study the genomics of vertebrate evo-devo. *J. Exp. Zoo. B Mol. Dev. Evol.* **324**, 316–341 (2015).

3. McCluskey, B. M. & Braasch, I. Chapter 2 - Zebrafish Phylogeny and Taxonomy. in *The Zebrafish in Biomedical Research* (eds. Cartner, S. C., Eisen, J.S., Farmer, S. C., Guillemin, K. J., Kent, M. L., & Sanders, G. E.) 15–24 (Academic Press, 2020) https://doi.org/10.1016/B978-0-12-812431-4.00002-6.

4. Herberg, S., Gert, K. R., Schleiffer, A. & Pauli, A. The Ly6/uPAR protein Bouncer is necessary and sufficient for species-specific fertilization. *Science* **361**, 1029–1033 (2018).

5. Tunyasuvunakool, K. *et al.* Highly accurate protein structure prediction for the human proteome. *Nature* **596**, 590–596 (2021).

6. Jumper, J. *et al.* Highly accurate protein structure prediction with AlphaFold. *Nature* **596**, 583–589 (2021).
