# Supplementary material for: Divergent molecular signatures in fish Bouncer proteins define cross-fertilization boundaries
Source: Nat Commun. 2023 Jun 14;14:3506. doi: 10.1038/s41467-023-39317-4 (PMC10267171; doi:10.1038/s41467-023-39317-4)
Supplement: Supplementary file 3 — Reporting Summary [file 41467_2023_39317_MOESM3_ESM.pdf]

nature portfolio

Corresponding author(s): Andrea Pauli

Last updated by author(s): Mar 12, 2023

Reporting Summary

Nature Portfolio wishes to improve the reproducibility of the work that we publish. This form provides structure for consistency and transparency in reporting. For further information on Nature Portfolio policies, see our Editorial Policies and the Editorial Policy Checklist.

Statistics

For all statistical analyses, confirm that the following items are present in the figure legend, table legend, main text, or Methods section.

n/a	Confirmed	
☐	☒	The exact sample size (*n*) for each experimental group/condition, given as a discrete number and unit of measurement
☐	☒	A statement on whether measurements were taken from distinct samples or whether the same sample was measured repeatedly
☐	☒	The statistical test(s) used AND whether they are one- or two-sided<br>*Only common tests should be described solely by name; describe more complex techniques in the Methods section.*
☒	☐	A description of all covariates tested
☐	☒	A description of any assumptions or corrections, such as tests of normality and adjustment for multiple comparisons
☐	☒	A full description of the statistical parameters including central tendency (e.g. means) or other basic estimates (e.g. regression coefficient) AND variation (e.g. standard deviation) or associated estimates of uncertainty (e.g. confidence intervals)
☐	☒	For null hypothesis testing, the test statistic (e.g. *F*, *t*, *r*) with confidence intervals, effect sizes, degrees of freedom and *P* value noted<br>*Give P values as exact values whenever suitable.*
☒	☐	For Bayesian analysis, information on the choice of priors and Markov chain Monte Carlo settings
☒	☐	For hierarchical and complex designs, identification of the appropriate level for tests and full reporting of outcomes
☒	☐	Estimates of effect sizes (e.g. Cohen's *d*, Pearson's *r*), indicating how they were calculated

Our web collection on statistics for biologists contains articles on many of the points above.

Software and code

Policy information about availability of computer code

Data collection

Mature Bncr protein sequences were aligned using MAFFT (Katoh et al., 2013) and codon alignment was generated using PAL2NAL (Suyama et al., 2006). Codon alignments were then used as input into IQ-TREE (Nguyen et al., 2015) to generate the best substitution model and a maximum-likelihood tree was generated using 1000 ultrafast bootstrap iterations (Hoang et al., 2018). Codon alignments and the maximum-likelihood tree were used as input into HyPhy (Pond et al., 2005) to test the mode of selection acting on Bncr in fish. A suite of tests was performed across all sequences by using MEME (Murrell et al., 2012), FUBAR (Murrell et al., 2013), FEL (Pond et al., 2005), and BUSTED (Murrell et al., 2015). The mode of selection acting on the zebrafish and medaka lineages was tested by selecting on the branches leading to these lineages and performing aBSREL (Smith et al., 2015) and Contrast-FEL (Pond et al., 2020). We further mapped the residues identified to be under selection regimes of interest onto the predicted 3-D structures of zebrafish and medaka Bncr (Jumper et al., 2021; Tunyasuvunakool, et al., 2021). In addition, the level of conservation was mapped onto the 3-D structures for zebrafish and medaka Bncr using CONSURF (Ashkenazy et al., 2016) and visualized using PyMOL (http://www.pymol.org).
For Bouncer ancestral state predictions, Bncr amino acid sequences were aligned using MUSCLE (Edgar et al., 2004) with default parameters. A phylogeny was reconstructed using MrBayes and mcmc=2000000 (Ronquist & Huelsenbeck, 2003). Ancestral amino acid states were reconstructed for all nodes of the obtained phylogeny with PAML with default parameters (Yang, 2007). The alignment, phylogeny, and ancestral reconstructions as well as the relevant control files are available on GitHub (https://github.com/kristabriedis/AncestralBncrs).
Bncr amino acid sequence algnments were visualized in JalView (version 2.10.5).
Fluorescent expression and localization of Bncr variants in transgenic eggs was assessed using CellMask Deep Red plasma membrane stain (Invitrogen; C10046); transgenic eggs were imaged using an upright point laser scanning confocal microscope (LSM800 Examiner Z1, Zeiss) with a 10X/0.3 N-achroplan water objective, using the Zeiss ZEN Blue software.
Enhanced chemiluminescence images of Western blots were recorded on a ChemiDoc (BioRad). Images were processed in FIJI (version 2.9.0).

Data analysis

Structural protein models were visualized in PyMOL 2.5.4.
Graphs and statistical analysis were generated with GraphPad Prism version 9.5.0 (525).

For manuscripts utilizing custom algorithms or software that are central to the research but not yet described in published literature, software must be made available to editors and reviewers. We strongly encourage code deposition in a community repository (e.g. GitHub). See the Nature Portfolio guidelines for submitting code & software for further information.

Data

Policy information about availability of data

All manuscripts must include a data availability statement. This statement should provide the following information, where applicable:

- Accession codes, unique identifiers, or web links for publicly available datasets
- A description of any restrictions on data availability
- For clinical datasets or third party data, please ensure that the statement adheres to our policy

Files pertaining to ancestral state reconstruction are available on GitHub (https://github.com/kristabriedis/AncestralBncrs).
All biological materials (mutant zebrafish lines or plasmids generated as part of this study) can be obtained from the corresponding author without any restrictions.

Human research participants

Policy information about studies involving human research participants and Sex and Gender in Research.

Reporting on sex and gender: n.a.

Population characteristics: n.a.

Recruitment: n.a.

Ethics oversight: n.a.

Note that full information on the approval of the study protocol must also be provided in the manuscript.

Field-specific reporting

Please select the one below that is the best fit for your research. If you are not sure, read the appropriate sections before making your selection.

☒ Life sciences ☐ Behavioural & social sciences ☐ Ecological, evolutionary & environmental sciences

For a reference copy of the document with all sections, see nature.com/documents/nr-reporting-summary-flat.pdf

Life sciences study design

All studies must disclose on these points even when the disclosure is negative.

Sample size

Sample sizes are described in the figure legends. No statistical calculations were done to predetermine the sample size. Sample sizes were chosen to be as large as possible while still feasible in terms of sample handling and data collection, and under consideration of the 3R rule to minimize the number of animals used to the number necessary to obtain conclusive results.

Data exclusions

For comparative in vitro fertilization (IVF) experiments using zebrafish versus medaka sperm, each species' sperm was tested with conspecific eggs to ensure the quality of the sperm and to confirm that no cross-species sperm contamination had occurred during sperm collection. If the sperm was found to be cross-contaminated, the entire experiment was excluded. Otherwise, individual samples were only excluded if fertilization rates were markedly poor compared to other rates obtained from the same individual; this was usually due to poor egg quality or abnormal cleavages such that fertilization rate could not be accurately counted.
For bias analyses, experimental datasets for which all values were equal to 0 were not used to calculate bias. For imaging experiments, eggs that had moved during imaging such that the membrane was obscured or such that they were no longer in focus were not used to assess GFP-Bncr localization.

Replication

Each condition (genotype) was tested at least in biological triplicates for at least 2 (in most cases at least 5) independent transgenic fish. Replication of experimental data was successful. The number of independent biological replicates are specified in the figure legend for each figure panel.

Randomization

Samples were grouped based on their genotypes or species of origin (WT or control rescue transgene versus different transgene variants; zebrafish versus medaka sperm). In case of the analysis of comparative fertilization rates between zebrafish and medaka sperm, each clutch of eggs was split in half and fertilized with either zebrafish or medaka sperm.

Blinding

Blinding was not possible during data collection since according to our animal protocols, fish and embryos need to be correctly labeled with their genotype and date of birth. However, after sample collection, all samples were treated equally and processed at the same time to

minimize possible biases introduced during unequal sample preparations. Fertilization rates were collected by counting fertilized eggs, which does not include subjective criteria that could influence the result.

Reporting for specific materials, systems and methods

We require information from authors about some types of materials, experimental systems and methods used in many studies. Here, indicate whether each material, system or method listed is relevant to your study. If you are not sure if a list item applies to your research, read the appropriate section before selecting a response.

Materials & experimental systems

n/a	Involved in the study
☐	☒ Antibodies
☒	☐ Eukaryotic cell lines
☒	☐ Palaeontology and archaeology
☐	☒ Animals and other organisms
☒	☐ Clinical data
☒	☐ Dual use research of concern

Methods

n/a	Involved in the study
☒	☐ ChIP-seq
☒	☐ Flow cytometry
☒	☐ MRI-based neuroimaging

Antibodies

Antibodies used	Primary antibodies: anti-GFP (rabbit, 1:1.000, Invitrogen A11122) and anti-alpha-Tubulin (mouse, 1:20.000, Merck T6074). Secondary antibodies: goat F(ab')2 anti-rabbit IgG (H+L)-HRPO (1:10.000, 111-036-045, Dianova), goat F(ab')2 anti-mouse IgG (H+L)-HRPO (1:10.000, 115-036-062, Dianova)
Validation	Only commercially available antibodies were used that had been confirmed to be specific by the manufacturer and were tested again by us beforehand for their specificity by Western Blotting.

Animals and other research organisms

Policy information about studies involving animals; ARRIVE guidelines recommended for reporting animal research, and Sex and Gender in Research

Laboratory animals	TLAB zebrafish, generated by crossing zebrafish AB with the natural variant TL (Tupfel Longfin), served as wild-type zebrafish for all experiments. Wild-type medaka (Oryzias latipes, CAB strain) were raised according to standard protocols and served as wild-type medaka. Oryzias curvinotus and F1 hybrids of O. curvinotus x O. latipes were raised under the same conditions. Bouncer mutant zebrafish have been published previously (Herberg et al., 2018). Adult fish ranged in the age from 3 months to 1.5 years.
Wild animals	The study did not involve wild animals.
Reporting on sex	Collection of fertilization rates requires in-crosses of males and females or collection of sperm and eggs for subsequent in vitro fertilization experiments. Males were used to collect sperm, females were used to collect eggs.
Field-collected samples	The study did not involve samples collected in the field.
Ethics oversight	All animal experiments were conducted according to Austrian and European guidelines for animal research and approved by local Austrian authorities ('Amt der Wiener Landesregierung, Magistratsabteilung 58 – Wasserrecht'): Animal protocols GZ 342445/2016/12 and MA 58-221180-2021-16 for work with zebrafish; animal protocol GZ: 198603/2018/14 for work with medaka.

Note that full information on the approval of the study protocol must also be provided in the manuscript.
