# Supplementary material for: Divergent molecular signatures in fish Bouncer proteins define cross-fertilization boundaries
Source: Nat Commun. 2023 Jun 14;14:3506. doi: 10.1038/s41467-023-39317-4 (PMC10267171; doi:10.1038/s41467-023-39317-4)
Supplement: Supplementary file 4 — Description to Additional Supplementary Information [file 41467_2023_39317_MOESM4_ESM.pdf]

Description of Additional Supplementary Files

File Name: Supplementary Data 1

Description: Wild-type and mutant medaka bncra and bncrb sequences.

File Name: Supplementary Data 2

Description: Bncr protein sequences in alignment shown in Figure 1B.

File Name: Supplementary Data 3

Description: Transgenic line Bncr sequences.

File Name: Supplementary Data 4

Description: Selection analyses of medaka and zebrafish Bncr.

File Name: Supplementary Data 5

Description: Predicted Bncr protein sequences of Russian sturgeon, American paddlefish, and Lake Victoria cichlids from NCBI/Ensembl databases
