# Supplementary material for: Divergent molecular signatures in fish Bouncer proteins define cross-fertilization boundaries
Source: Nat Commun. 2023 Jun 14;14:3506. doi: 10.1038/s41467-023-39317-4 (PMC10267171; doi:10.1038/s41467-023-39317-4)
Supplement: Supplementary file 5 — Supplementary Data File 1 [file 41467_2023_39317_MOESM5_ESM.pdf]

Supplementary Data File 1

Wild-type and mutant medaka *bncra* and *bncrb* sequences

Exons are annotated in the following color code: exon 1, exon 2, exon 3

Wild-type *bncra* cDNA; deletion indicated in <u>bold</u>
ATGGGATCACTGAGAACCAGGCAGCTCTTCCATGCTGCTTTGCTGTGGCTTTGCCTTCC
CCTTCCTCTGCTGCTCTGTGAAAACCTGCATTGCTACTACAGCCCC**<u>GTCCT</u>**GGAGAAG
GAAATAACGTTTGAACTCGTCGTGACAGAATGCCCTCCGAATGAGATGTGCTTTAAGGG
GTTGGGTCGCTACGGCAACTACACTGCCCTATCAGCCAGGGGCTGCATGTTGGAGAAA
GACTGCAGTCAGGTTCACAGCCTACGTCTCCTGGGCACCGTCTACACCATGAGCTACA
GCTGCTGTGACTGGCCGTACTGTAACCGGGCCGTCGCCCTGGAGCCGCTCACTGCTA
TGCTGGTGGCTGCTGCTGTGGTGGCCTGCAGCTTTTGTCTAACATGA

Wild-type *bncra* cDNA translation (131 amino acids)
MGSLRTRQLFHAALLWLCLPLPLLLCENLHCYYSPVLEKEITFELVVTECPPNEMCFKGLGR
YGNYTALSARGCMLEKDCSQVHSLRLLGTVYTMSYSCCDWPYCNRAVALEPLTAMLVAAA
VVACSFCLT*

Mutant *bncra* (5-nt deletion) cDNA
ATGGGATCACTGAGAACCAGGCAGCTCTTCCATGCTGCTTTGCTGTGGCTTTGCCTTCC
CCTTCCTCTGCTGCTCTGTGAAAACCTGCATTGCTACTACAGCCCCGGAGAAGGAAATA
ACGTTTGA

Mutant *bncra* (5-nt deletion) cDNA translation (41 amino acids)
MGSLRTRQLFHAALLWLCLPLPLLLCENLHCYYSPGEGNNV*

Wild-type *bncrb* cDNA; deletion indicated in <u>bold</u>
ATGGGATCACTGAGAACCAGCACAATTTTGGGCCAACCTCAATTGCTCCTCGCCTCCAT
CCTGTTGGTTTCTGGTCCCCTCAGTCCTGTCTCTA**<u>CCCTGGAACACCTCTTGTGTAACG</u>**
<u>TCTGCCCCCTGCAT</u>GAAAAATCTGAGTTGTGTCCAAACTTCACCACCGAGTGTCGGCC
CGGCGAGCGCTGCACCAGCTCAAGAGGCTTCTACGGTGCCCTTCACGTCCTTTCCGCT
CAGGGCTGCATCAGTGCCGACCTCTGTGGTTCCTATGAGATGGTCACTTACAGAGGAA
TCAAATATAAACTTCGTTATGCTTGCTGCTGCGGAAACACATGTAACGAGGCGCCTGAA
TCCAAAACCACACTGAAGGAGCTGCTGCAGATGATCCAAGCTAAAGCAAATGGCACTG
AGGCTGCTGTGGAAAAGCCTTTGGCTGTGTGTGCAAACAACACACTGATAGAAACCAG
TGCTCCTCCTGCTGTTAAGGCATAG

Wild-type *bncrb* cDNA translation (163 amino acids)
MGSLRTSTILGQPQLLLASILLVSGPLSPVSTLEHLLCNVCPLHEKSELCPNFTTECRPGERC
TSSRGFYGALHVLSAQGCISADLCGSYEMVTYRGIKYKLRYACCCGNTCNEAPESKTTLKE
LLQMIQAKANGTEAAVEKPLAVCANNTLIETSAPPAVKA*

Mutant *bncrb* (38-nt deletion) cDNA
ATGGGATCACTGAGAACCAGCACAATTTTGGGCCAACCTCAATTGCTCCTCGCCTCCAT
CCTGTTGGTTTCTGGTCCCCTCAGTCCTGTCTCTAGAAAAATCTGA

Mutant *bncrb* (38-nt deletion) cDNA translation (34 amino acids)
MGSLRTSTILGQPQLLLASILLVSGPLSPVSRKI*
