# Supplementary material for: Divergent molecular signatures in fish Bouncer proteins define cross-fertilization boundaries
Source: Nat Commun. 2023 Jun 14;14:3506. doi: 10.1038/s41467-023-39317-4 (PMC10267171; doi:10.1038/s41467-023-39317-4)
Supplement: Supplementary file 6 — Supplementary Data File 2 [file 41467_2023_39317_MOESM6_ESM.pdf]

Supplementary Data File 2
Bncr protein sequences in alignment shown in Figure 1B.

>Danio_rerio_XP_005173770.1 (zebrafish Bncr)
QGLRCLFCPVTSLNSSCAPVVTECPVQELCYTADGRFGRSSVLFRKGCMLRADCSRSRHQ
MIRGNNISFSFSCCGGHYCN

>Oryzias_latipes_H2LID1.1 (medaka Bncra)
ENLHCYYSPVLEKEITFELVVTECPPNEMCFKGLGRYGNYTALSARGCMLEKDCSQVHSLR
LLGTVYTMSYSCCDWPYCN

>Oryzias_latipes_H2LID5 (medaka Bncrb)
EHLLCNVCPLHEKSELCPNFTTECRPGERCTSSRGFYGALHVLSAQGCISADLCGSYEMVT
YRGIKYKLRYACCCGNTCN

>Cyprinus_carpio_XP_018955736.2 (carp Bncra)
ENLYCYYCPQTSFNRSCRHILSECRPQELCFTALGRFGHAPVLFSKGCMSQRDCVRSSSQ
MIRGNNISFTNSCCGRPYCN

>Cyprinus_carpio_XP_018955710.2 (carp Bncrb)
VLLCHYCPLQAAGTRCNITTECLEHERCSSGWRRYGRVHVLALQGCLSPELCGSNQTLTH
KGLEYEITYTCCCRDLCN

>Takifugu_rubripes_XP_011605859.1 (fugu Bncra)
DNLLCYFSPLLEKEVSFKFIATECPPGDLCFKADGRYGNHSALSGRGCMAREACSQTHSIR
YKGSVFVMSYSCCDSPYCN

>Takifugu_rubripes_XP_011605858.1 (fugu Bncrb)
DTLLCYFCPLQHKTDSCVNTTSRCPPTQRCSSSRGHYGLVHVLSAQGCMDVALCGSYEIL
SFKGTDFNVSHTCCCKDQCN

>Hippocampus_comes_XP_019712504.1 (seahorse Bncra)
GNLRCLYRPILEKEYEFQPIVTECPRGEVCYKAEGRYGNYSALSASGCMPRRVCGLQHDL
SYQGVVYTMSYSCCDRPYCN

>Hippocampus_comes_translation from genomic frame 5KV880484.1_5 (seahorse Bncrb)
TSLLCHFCPLQPKEFPCTNLTTECMPGQRCATSRAYYGVVHVLSAQGCVDARLCGNRLSV
SHMGVEYRLRHSCCCKDKCN

>Salmo_salar_XP_013981439.1 (salmon Bncra)
NNLLCYYSPIMYRNKTFDLILTECPPTELCMTGNGRYGNHSALSTRGCVAPTGCGQVHPLR
LKGTVYTMTYACCDYNYCN

>Salmo_salar_XP_013981440.1 (salmon Bncrb)
TSLRCNFCPLQHKGRSCSNDSTTECLPQERCGTSSGRFGPIHILSAQGCLTPDLCNSTHAV
TYRGVSYNVTYRCCCRDQCN
