# Supplementary material for: Divergent molecular signatures in fish Bouncer proteins define cross-fertilization boundaries
Source: Nat Commun. 2023 Jun 14;14:3506. doi: 10.1038/s41467-023-39317-4 (PMC10267171; doi:10.1038/s41467-023-39317-4)
Supplement: Supplementary file 7 — Supplementary Data File 3 [file 41467_2023_39317_MOESM7_ESM.pdf]

Supplementary Data File 3
Transgenic line Bncr sequences

All Bncr protein sequences listed below are preceded by the zebrafish Bncr signal peptide sequence and sfGFP ORF (no stop codon) as follows:

MGCVLLFLLLVCVPVVLPTRVSKGEELFTGVVPILVELDGDVNGHKFSVRGEGEGDATNGK
LTLKFICTTGKLPVPWPTLVTTLTYGVQCFSRYPDHMKQHDFFKSAMPEGYVQERTISFKD
DGTYKTRAEVKFEGDTLVNRIELKGIDFKEDGNILGHKLEYNFNSHNVYITADKQKNGIKANF
KIRHNVEDGSVQLADHYQQNTPIGDGPVLLPDNHYLSTQSVLSKDPNEKRDHMVLLEFVTA
AGITLGMDELYKTRAAEF…

Other fish Bncr homologs

Medaka Bncra
…ENLHCYYSPVLEKEITFELVVTECPPNEMCFKGLGRYGNYTALSARGCMLEKDCSQVHS
LRLLGTVYTMSYSCCDWPYCNRAVALEPFTAMLVAAAVVACSFCLT*

Medaka Bncrb
…LEHLLCNVCPLHEKSELCPNFTTECRPGERCTSSRGFYGALHVLSAQGCISADLCGSYE
MVTYRGIKYKLRYACCCGNTCNEAPESKTTLKELLQMIQAKANGTEAAVEKPLAVCANNTLI
ETSAPPAVKA*

Carp Bncra
…ENLYCYYCPQTSFNRSCRHILSECRPQELCFTALGRFGHAPVLFSKGCMSQRDCVRSSS
QMIRGNNISFTNSCCGRPYCNSSRGCDHSLALLTVSAITASVLTADWTRAGLMMPS*

Seahorse Bncra
…GNLRCLYRPILEKEYEFQPIVTECPRGEVCYKAEGRYGNYSALSASGCMPRRVCGLQHD
LSYQGVVYTMSYSCCDRPYCNACVGLFANTLVITVTLVTVAGMVGR*

Fugu Bncra
…DNLLCYFSPLLEKEVSFKFIATECPPGDLCFKADGRYGNHSALSGRGCMAREACSQTHSI
RYKGSVFVMSYSCCDSPYCNSCPGVAAPPFCIAAALLTAALITSPRDVLRGVFSFILE*

Chimeric Bncr sequences
All sequences are derived from zebrafish Bncr except amino acids in bold which are changed to the medaka Bncr sequence and named accordingly.

Medaka base
…**ENLHC**LFCPVTSLNSSCAPVVTE**CPPNEMC**YTADGRFGRSSVLFRKG**CMLEKDC**SRSR
HQMIRGNNISFSFS**CCDWPYCNRAVALEPFTAMLVAAAVVACSFCLT***

Medaka top
…QGLRC**YYSPVLEKEITFELVVTE**CPVQELC**FKGLGRYGNYTALSARG**CMLRADC**SQVH
SLRLLGTVYTMSYS**CCGGHYCNSQPRAEPGGRLLLLLLPAAALTAAGAL*

Medaka finger 1
…QGLRC**YYSPVLEKEITFELVVTE**CPVQELCYTADGRFGRSSVLFRKGCMLRADCSRSRH
QMIRGNNISFSFSCCGGHYCNSQPRAEPGGRLLLLLLPAAALTAAGAL*

Medaka finger 2
…QGLRCLFCPVTSLNSSCAPVVTECPVQELC**FKGLGRYGNYTALSARG**CMLRADCSRSR
HQMIRGNNISFSFSCCGGHYCNSQPRAEPGGRLLLLLLPAAALTAAGAL*

Medaka finger 3
…QGLRCLFCPVTSLNSSCAPVVTECPVQELCYTADGRFGRSSVLFRKGCMLRADC**SQVH**
SLRLLGTVYTMSYSCCGGHYCNSQPRAEPGGRLLLLLLPAAALTAAGAL*

Medaka fingers 1 + 2
…QGLRC**YYSPVLEKEITFELVVTE**CPVQELC**FKGLGRYGNYTALSARG**CMLRADCSRSR
HQMIRGNNISFSFSCCGGHYCNSQPRAEPGGRLLLLLLPAAALTAAGAL*

Medaka fingers 1 + 3
…**ENLHCYYSPVLEKEITFELVVTECPPNEMC**YTADGRFGRSSVLFRKG**CMLEKDCSQVHS**
LRLLGTVYTMSYSCCDWPYCNRAVALEPFTAMLVAAAVVACSFCLT*

Medaka fingers 2 + 3
…QGLRCLFCPVTSLNSSCAPVVTECPVQELC**FKGLGRYGNYTALSARG**CMLRADC**SQVH**
SLRLLGTVYTMSYSCCGGHYCNSQPRAEPGGRLLLLLLPAAALTAAGAL*

<u>Ancestral state sequences</u>

Nodes A-D
…DNLRCYYSPILEKEKTFELIVTECPPDELCFKADGRYGNHSALSARGCMAKKDCGQVHKL
RLKGTVYTMSYSCCDWPYCNSQPRAEPGGRLLLLLLPAAALTAAGTL*

Node E
…ENLHCYYSPILEKEKTFELIVTECPPNELCFKALGRYGNYTALSARGCMPEKDCSQVHNL
RLRGTVYTMSYSCCDWPYCNSQPRAEPGGRLLLLLLPAAALTAAGTL*

Node F
…ENLHCYYSPILEKEITFELIVTECPPNELCFKALGRYGNYTALSARGCMLEKDCSQVHSLR
LLGTVYTMSYSCCDWPYCNSQPRAEPGGRLLLLLLPAAALTAAGTL*

Node G
…DNLRCYYSPILEKEKTFELIVTECPPDELCFKADGRYGNHSALSARGCMAKKDCGQVHKL
RFKGTVYTMSYACCDGPYCNSQPRAEPGGRLLLLLLPAAALTAAGTL*

<u>Amino acid substitution sequences</u>
Substitution mutations are marked in bold.

Medaka Bncra I15S
…ENLHCYYSPVLEKE**S**TFELVVTECPPNEMCFKGLGRYGNYTALSARGCMLEKDCSQVHS
LRLLGTVYTMSYSCCDWPYCNRAVALEPFTAMLVAAAVVACSFCLT*

Medaka Bncra L63R
…ENLHCYYSPVLEKEITFELVVTECPPNEMCFKGLGRYGNYTALSARGCMLEKDCSQVHS
LRL**R**GTVYTMSYSCCDWPYCNRAVALEPFTAMLVAAAVVACSFCLT*

Medaka Bncra I15S, L63R
…ENLHCYYSPVLEKE**S**TFELVVTECPPNEMCFKGLGRYGNYTALSARGCMLEKDCSQVHS
LRL**R**GTVYTMSYSCCDWPYCNRAVALEPFTAMLVAAAVVACSFCLT*

Medaka Bncra I15S, A45R
…ENLHCYYSPVLEKE**S**TFELVVTECPPNEMCFKGLGRYGNYTALS**R**RGCMLEKDCSQVH
SLRLLGTVYTMSYSCCDWPYCNRAVALEPFTAMLVAAAVVACSFCLT*

Medaka Bncra A45R, L63R

…ENLHCYYSPVLEKEITFELVVTECPPNEMCFKGLGRYGNYTALS**R**RGCMLEKDCSQVHS
LRL**R**GTVYTMSYSCCDWPYCNRAVALEPFTAMLVAAAVVACSFCLT*

Medaka Bncra I15S, A45R, L63R
…ENLHCYYSPVLEKE**S**TFELVVTECPPNEMCFKGLGRYGNYTALS**R**RGCMLEKDCSQVH
SLRL**R**GTVYTMSYSCCDWPYCNRAVALEPFTAMLVAAAVVACSFCLT*
Zebrafish Bncr S15I
…QGLRCLFCPVTSLN**I**SCAPVVTECPVQELCYTADGRFGRSSVLFRKGCMLRADCSRSRH
QMIRGNNISFSFSCCGGHYCNSQPRAEPGGRLLLLLLPAAALTAAGAL*

Zebrafish Bncr R63L
…QGLRCLFCPVTSLNSSCAPVVTECPVQELCYTADGRFGRSSVLFRKGCMLRADCSRSR
HQMI**L**GNNISFSFSCCGGHYCNSQPRAEPGGRLLLLLLPAAALTAAGAL*

Zebrafish Bncr S15I, R63L
…QGLRCLFCPVTSLN**I**SCAPVVTECPVQELCYTADGRFGRSSVLFRKGCMLRADCSRSRH
QMI**L**GNNISFSFSCCGGHYCNSQPRAEPGGRLLLLLLPAAALTAAGAL*

Zebrafish Bncr S15I, R45A
…QGLRCLFCPVTSLN**I**SCAPVVTECPVQELCYTADGRFGRSSVLF**A**KGCMLRADCSRSRH
QMIRGNNISFSFSCCGGHYCNSQPRAEPGGRLLLLLLPAAALTAAGAL*

Zebrafish Bncr R45A, R63L
…QGLRCLFCPVTSLNSSCAPVVTECPVQELCYTADGRFGRSSVLF**A**KGCMLRADCSRSR
HQMI**L**GNNISFSFSCCGGHYCNSQPRAEPGGRLLLLLLPAAALTAAGAL*

Zebrafish Bncr S15I, R45A, R63L
…QGLRCLFCPVTSLN**I**SCAPVVTECPVQELCYTADGRFGRSSVLF**A**KGCMLRADCSRSRH
QMI**L**GNNISFSFSCCGGHYCNSQPRAEPGGRLLLLLLPAAALTAAGAL*

N-glycosylation variant sequences
N-glycosylation site mutations are marked in bold; the three amino acids of one species' Bncr were changed to the corresponding three amino acids of the other species' Bncr to either introduce or remove each N-glycosylation consensus sequence.

Zebrafish Bncr +glyc2
…QGLRCLFCPVTSLNSSCAPVVTECPVQELCYTADGRFG**NYT**VLFRKGCMLRADCSRSR
HQMIRGNNISFSFSCCGGHYCNSQPRAEPGGRLLLLLLPAAALTAAGAL*

Zebrafish Bncr +glyc2, -glyc3
…QGLRCLFCPVTSLNSSCAPVVTECPVQELCYTADGRFG**NYT**VLFRKGCMLRADCSRSR
HQMIRGN**VYT**FSFSCCGGHYCNSQPRAEPGGRLLLLLLPAAALTAAGAL*

Zebrafish Bncr +glyc2, -glyc1
…QGLRCLFCPVTSL**EIT**CAPVVTECPVQELCYTADGRFG**NYT**VLFRKGCMLRADCSRSRH
QMIRGNNISFSFSCCGGHYCNSQPRAEPGGRLLLLLLPAAALTAAGAL*

Zebrafish Bncr +glyc2, -glyc(1+3)
…QGLRCLFCPVTSL**EIT**CAPVVTECPVQELCYTADGRFG**NYT**VLFRKGCMLRADCSRSRH
QMIRGN**VYT**FSFSCCGGHYCNSQPRAEPGGRLLLLLLPAAALTAAGAL*

Medaka Bncra -glyc2
…ENLHCYYSPVLEKEITFELVVTECPPNEMCFKGLGRYG**RSS**ALSARGCMLEKDCSQVHS
LRLLGTVYTMSYSCCDWPYCNRAVALEPFTAMLVAAAVVACSFCLT*

Medaka Bncra -glyc2, +glyc1
…ENLHCYYSPVLEK**NSS**FELVVTECPPNEMCFKGLGRYG**RSS**ALSARGCMLEKDCSQVH
SLRLLGTVYTMSYSCCDWPYCNRAVALEPFTAMLVAAAVVACSFCLT*

Medaka Bncra -glyc2, +glyc3
…ENLHCYYSPVLEKEITFELVVTECPPNEMCFKGLGRYG**RSS**ALSARGCMLEKDCSQVHS
LRLLGT**NIS**MSYSCCDWPYCNRAVALEPFTAMLVAAAVVACSFCLT*

Medaka Bncra -glyc2, +glyc(1+3)
…ENLHCYYSPVLEK**NSS**FELVVTECPPNEMCFKGLGRYG**RSS**ALSARGCMLEKDCSQVH
SLRLLGT**NIS**MSYSCCDWPYCNRAVALEPFTAMLVAAAVVACSFCLT*

<u>N-glycosylation/amino acid variant combination sequences</u>
N-glycosylation consensus sequence mutations and amino acid substitutions are marked in bold.

Medaka Bncra -glyc2, +glyc(1+3), L63R
…ENLHCYYSPVLEK**NSS**FELVVTECPPNEMCFKGLGRYG**RSS**ALSARGCMLEKDCSQVH
SLRL**R**GT**NIS**MSYSCCDWPYCNRAVALEPFTAMLVAAAVVACSFCLT*

Medaka Bncra -glyc2, +glyc(1+3), A45R, L63R
…ENLHCYYSPVLEK**NSS**FELVVTECPPNEMCFKGLGRYG**RSS**ALS**R**RGCMLEKDCSQVH
SLRL**R**GT**NIS**MSYSCCDWPYCNRAVALEPFTAMLVAAAVVACSFCLT*

Zebrafish Bncr, +glyc2, -glyc(1+3), R63L
…QGLRCLFCPVTSL**EIT**CAPVVTECPVQELCYTADGRFG**NYT**VLFRKGCMLRADCSRSRH
QMI**L**GN**VYT**FSFSCCGGHYCNSQPRAEPGGRLLLLLLPAAALTAAGAL*

Zebrafish Bncr, +glyc2, -glyc(1+3), R45A, R63L
…QGLRCLFCPVTSL**EIT**CAPVVTECPVQELCYTADGRFG**NYT**VLF**A**KGCMLRADCSRSRH
QMI**L**GN**VYT**FSFSCCGGHYCNSQPRAEPGGRLLLLLLPAAALTAAGAL*

Zebrafish Bncr, +glyc2, medaka finger 3
…QGLRCLFCPVTSLNSSCAPVVTECPVQELCYTADGRFG**NYT**VLFRKGCMLRADC**SQVH**
SLRLLGTVYTMSYSCCGGHYCNSQPRAEPGGRLLLLLLPAAALTAAGAL*
