# Supplementary material for: Divergent molecular signatures in fish Bouncer proteins define cross-fertilization boundaries
Source: Nat Commun. 2023 Jun 14;14:3506. doi: 10.1038/s41467-023-39317-4 (PMC10267171; doi:10.1038/s41467-023-39317-4)
Supplement: Supplementary file 8 — Supplementary Data File 4 [file 41467_2023_39317_MOESM8_ESM.pdf]

Supplementary Data File 4

Selection analyses of medaka and zebrafish Bncr

1	Positive selection	-1	Negative selection

Codon site in alignment	MEME	MEME number of branches under selection	FUBAR	FEL all	FEL Danio branch	FEL Oryzias branch	FEL Contrast Danio vs. Oryzias	BUSTED all	Danio rerio AA in alignment	Oryzias latipes AA in alignment	Danio rerio site	Oryzias latipes site
1	0	0	-1	-1	0	0	0	0	Q	E	1	1
2	0	0	-1	-1	0	0	0	0	G	N	2	2
3	1	2	0	0	0	0	0	0	L	L	3	3
4	1	1	-1	-1	0	0	0	0	R	H	4	4
5	0	1	-1	-1	-1	0	0	0	C	C	5	5
6	0	0	0	0	0	0	0	0	L	Y	6	6
7	0	1	0	0	0	0	0	0	F	Y	7	7
8	0	0	-1	-1	-1	0	0	0	C	S	8	8
9	0	1	-1	-1	-1	0	0	0	P	P	9	9
10	0	0	0	0	0	0	0	0	V	V	10	10
11	0	0	0	0	0	0	0	0	T	L	11	11
12	0	0	0	0	0	0	0	0	S	E	12	12
13	0	0	0	0	0	0	0	0	-	-	12	12
14	0	1	0	0	0	0	0	0	-	-	12	12
15	0	0	-1	-1	0	0	0	0	L	K	13	13
16	0	0	0	0	0	0	0	0	N	E	14	14
17	1	6	0	1	1	1	0	0	S	I	15	15
18	0	1	-1	-1	0	0	0	0	S	T	16	16
19	0	3	-1	-1	-1	0	0	0	C	F	17	17
20	0	0	0	0	0	0	0	0	A	E	18	18
21	0	0	-1	-1	0	0	0	0	P	L	19	19
22	0	4	0	-1	-1	0	0	0	V	V	20	20
23	0	0	0	0	0	0	0	0	V	V	21	21
24	0	0	-1	-1	0	0	0	0	T	T	22	22
25	0	2	-1	-1	-1	0	0	0	E	E	23	23
26	0	0	-1	-1	-1	0	0	0	C	C	24	24
27	0	0	-1	-1	-1	0	0	0	P	P	25	25
28	0	0	-1	-1	0	0	0	0	V	P	26	26
29	0	0	-1	0	-1	0	0	0	Q	N	27	27
30	0	0	-1	0	0	0	0	0	E	E	28	28
31	0	0	0	0	0	0	0	0	L	M	29	29
32	0	0	0	0	0	0	0	0	-	-	29	29
33	0	0	-1	-1	0	0	0	0	C	C	30	30
34	0	0	-1	-1	-1	-1	0	0	Y	F	31	31
35	0	0	-1	-1	-1	0	0	0	T	K	32	32
36	0	0	0	0	0	0	0	0	-	-	32	32
37	0	0	0	0	0	0	0	0	-	-	32	32
38	0	0	0	0	0	0	0	0	-	-	32	32
39	0	0	0	0	0	0	0	0	-	-	32	32
40	0	5	-1	-1	-1	0	0	0	A	G	33	33
41	1	1	-1	-1	0	0	0	0	D	L	34	34
42	0	3	0	0	0	0	0	0	G	G	35	35
43	0	2	0	0	0	0	0	0	R	R	36	36
44	0	0	-1	-1	0	0	0	0	F	Y	37	37

45	0	0	-1	-1	-1	0	0	0	G	G	38	38
46	0	0	-1	-1	0	0	0	0	R	N	39	39
47	1	2	0	0	0	0	0	0	S	Y	40	40
48	0	0	-1	-1	0	0	0	0	S	T	41	41
49	0	0	0	-1	0	0	0	0	V	A	42	42
50	0	0	-1	-1	-1	0	0	0	L	L	43	43
51	0	0	-1	-1	0	0	0	0	F	S	44	44
52	0	0	-1	0	0	0	0	0	R	A	45	45
53	0	0	-1	-1	0	0	0	0	K	R	46	46
54	0	0	-1	-1	-1	0	0	0	G	G	47	47
55	0	0	-1	-1	-1	0	0	0	C	C	48	48
56	0	1	0	0	0	0	0	0	M	M	49	49
57	0	0	-1	0	0	1	0	0	L	L	50	50
58	0	0	0	0	0	0	0	0	R	E	51	51
59	0	0	-1	-1	0	0	0	0	A	K	52	52
60	1	6	0	0	0	0	0	0	D	D	53	53
61	0	0	-1	-1	-1	0	0	0	C	C	54	54
62	0	0	0	0	0	0	0	0	S	S	55	55
63	0	0	0	0	0	0	0	0	R	Q	56	56
64	0	0	-1	0	0	0	0	0	S	V	57	57
65	0	0	0	0	0	0	0	0	R	H	58	58
66	0	0	-1	-1	0	0	0	0	H	S	59	59
67	0	0	-1	-1	0	0	0	0	Q	L	60	60
68	0	0	-1	0	0	0	0	0	M	R	61	61
69	0	0	0	0	0	0	0	0	I	L	62	62
70	0	1	-1	-1	0	1	Oryzias positive	1	R	L	63	63
71	0	0	-1	-1	-1	0	0	0	G	G	64	64
72	0	0	-1	-1	0	0	0	0	N	T	65	65
73	0	0	-1	-1	-1	0	0	0	N	V	66	66
74	0	0	0	0	0	0	0	0	I	Y	67	67
75	0	0	0	0	0	0	0	0	S	T	68	68
76	0	0	0	0	0	0	0	0	F	M	69	69
77	0	0	0	-1	0	0	0	0	S	S	70	70
78	0	0	0	0	0	0	0	0	F	Y	71	71
79	0	0	0	0	0	0	0	0	S	S	72	72
80	0	0	-1	-1	0	0	0	0	C	C	73	73
81	0	0	-1	-1	-1	0	0	0	C	C	74	74
82	0	0	-1	-1	0	0	0	0	G	D	75	75
83	0	2	0	0	0	0	0	0	G	W	76	76
84	0	0	-1	-1	0	0	0	0	H	P	77	77
85	0	0	0	0	0	0	0	0	Y	Y	78	78
86	0	0	-1	-1	-1	0	0	0	C	C	79	79
87	0	0	-1	-1	-1	0	0	0	N	N	80	80
