# Supplementary material for: Divergent molecular signatures in fish Bouncer proteins define cross-fertilization boundaries
Source: Nat Commun. 2023 Jun 14;14:3506. doi: 10.1038/s41467-023-39317-4 (PMC10267171; doi:10.1038/s41467-023-39317-4)
Supplement: Supplementary file 9 — Supplementary Data File 5 [file 41467_2023_39317_MOESM9_ESM.pdf]

Supplementary Data File 5

Predicted Bncr protein sequences of Russian sturgeon, American paddlefish, and Lake Victoria cichlids from NCBI/Ensembl databases. Mature protein sequences are in bold.

>Acipenser_gueldenstaedtii_isolate M9 k119_2797981, whole genome shotgun sequence JANDEF011322014.1 (Russian sturgeon Bncr)
MSRLSSLLLCAAVLQQVVTSR**ALTCYYCQFDRKGRGCSNLQSQCVSGGACFTGAGLYGG LEVLKGKGCVDKELCNDLGSTDFRGVTYTVKYDCCRHDLCN**SRSEPSAGPSRVTLLMGV GVVLLRT*

>Polyodon_spathula_ XP_041112154.1 (American paddlefish Bncr)
MNRLSSLLLCTVLLQQIMSSG**ALTCYYCQFDRRERGCSNLQTQCVHGGACFAGSGLYGG LEVLKGKGCVDKELCNDLGSTDFRGVTYTVKYDCCRHDLCN**SRSELSARPARGTLLMAV GVVLFWFRT*

Haplochromine cichlid Bncrs

>Pundamilia_nyererei_XR_311598.2 (Makobe Island cichlid Bncr)
MLKLLHITGLWLHFLLPSVLC**DNLLCFYSPMLEKDKTPEFVVTECPPSKVCFMADGRYGNH SVLSARGCMAKKDCSQKQKVHFKGTTYTVSYSCCDQPHCN**SCLNIALEPLCLTLALVTVW VMVGDGL*

>Astatotilapia_calliptera_XR_003272940.1 (Eastern happy Bncr)
MLKLLHITGLWLHFLLPSVLC**DNLLCFYSPMLEKDKTPEFVVTECPPSKVCFMADGRYGNH SVLSARGCMAKKDCSQKQKVHFKGTTYTVSYSCCDQPHCN**SCLNIALEPLCLTLALVTVW VMVGDGL*

>Maylandia_zebra_predicted_Bncr
primary_assembly:M_zebra_UMD2a:LG7:15554292:15555131:-1 (Zebra mbuna Bncr)
MLKLLHITGLWLHFLLPSVLC**DNLLCFYSPMLEKDKTPEFVVTECPPSKVCFMADGRYGNH SVLSARGCMAKKDCSQKQKVHFKGTTYTVSYSCCDQPHCN**SFLNIALEPLCLTLALVTVW VMVGDGL*

>Haplochromis_burtoni_predicted_Bncr
primary_assembly:AstBur1.0:JH425424.1:933317:934156:1 (Burton’s mouthbrooder Bncr)
MLKLLHITGLWLHFLLPSVLC**DNLLCFYSPMLEKDKTPEFVVTECPPSKVCFMADGRYGNH SVLSARGCMAKKDCSQKQKVHFKGTTYTVSYSCCDQPHCN**SCLNIALEPLCLTLALVTVW VMVGDGL*
